# Three-Dimensional Biologically Relevant Spectrum (BRS-3D): Shape Similarity Profile Based on PDB Ligands as Molecular Descriptors

**DOI:** 10.3390/molecules21111554

**Published:** 2016-11-17

**Authors:** Ben Hu, Zheng-Kun Kuang, Shi-Yu Feng, Dong Wang, Song-Bing He, De-Xin Kong

**Affiliations:** 1 State Key Laboratory of Agricultural Microbiology, Huazhong Agricultural University, Wuhan 430070, China; benhu917@gmail.com (B.H.); kzk@webmail.hzau.edu.cn (Z.-K.K.); fshiyu@webmail.hzau.edu.cn (S.-Y.F.); duke.e.wang@gmail.com (D.W.); cencibaitai@webmail.hzau.edu.cn (S.-B.H.); 2 Agricultural Bioinformatics Key Laboratory of Hubei Province, College of Informatics, Huazhong Agricultural University, Wuhan 430070, China

**Keywords:** BRS-3D, molecular similarity profile, QSAR, SVM, ligand-based virtual screening, subtype selectivity

## Abstract

The crystallized ligands in the Protein Data Bank (PDB) can be treated as the inverse shapes of the active sites of corresponding proteins. Therefore, the shape similarity between a molecule and PDB ligands indicated the possibility of the molecule to bind with the targets. In this paper, we proposed a shape similarity profile that can be used as a molecular descriptor for ligand-based virtual screening. First, through three-dimensional (3D) structural clustering, 300 diverse ligands were extracted from the druggable protein–ligand database, sc-PDB. Then, each of the molecules under scrutiny was flexibly superimposed onto the 300 ligands. Superimpositions were scored by shape overlap and property similarity, producing a 300 dimensional similarity array termed the “Three-Dimensional Biologically Relevant Spectrum (BRS-3D)”. Finally, quantitative or discriminant models were developed with the 300 dimensional descriptor using machine learning methods (support vector machine). The effectiveness of this approach was evaluated using 42 benchmark data sets from the G protein-coupled receptor (GPCR) ligand library and the GPCR decoy database (GLL/GDD). We compared the performance of BRS-3D with other 2D and 3D state-of-the-art molecular descriptors. The results showed that models built with BRS-3D performed best for most GLL/GDD data sets. We also applied BRS-3D in histone deacetylase 1 inhibitors screening and GPCR subtype selectivity prediction. The advantages and disadvantages of this approach are discussed.

## 1. Introduction

Computer-aided drug discovery includes structure-based and ligand-based methods. Over the last few decades, advances in both scoring algorithm and computer capability have made structure-based drug discovery a popular tool in hit identification [1,2,3]. However, the massive publicly available libraries of bioactivity screening data are growing rapidly [4,5,6]. To exploit such big-data for drug discovery, ligand-based approaches are becoming increasingly important. According to how the molecular structure’s features are represented, ligand-based approaches can be categorized into two-dimensional (2D) or three-dimensional (3D) ones. The utility of the 2D approaches (based on 2D molecular descriptor or fingerprint) have been confirmed with a variety of algorithms, including similarity coefficients (e.g., Tanimoto coefficient), distance functions (e.g., Euclidean distance), or, most recently, the Similarity Ensemble Approach (SEA) algorithm [7].

In contrast to 2D methods, 3D methods (including 3D-QSAR [8,9] and pharmacophore modeling [10,11]) are based on molecular shape or conformation. As the ligand–receptor interaction involves 3D shape and properties (hydrophobic and electrostatic potential) complementarity, 3D methods are considered to have more potential for rational drug design, especially for scaffold hopping [12], even though previous studies show that 2D approaches are usually much faster and perform better than 3D ones [12,13,14]. Most 3D methods depend on hypothetical active conformations and require that all of the molecules are superimposed in advance. This situation is true for both CoMFA-related methods and pharmacophore approaches. Therefore, these traditional 3D methods are more applicable in situations when the active compounds share similar scaffolds or pharmacophores. However, if the active conformations were unavailable, the superimpositions were not feasible.

In 2011, we built a database using all the rigid active compounds in PubChem (unpublished work) with the expectation of guiding ligand design for corresponding targets. However, the disadvantages of weak activity and low molecular weight limited its application. In fact, our knowledge about the biologically active conformations is compiled as complexes with biological macromolecules in the Protein Data Bank (PDB; http://www.rcsb.org) [15]. The crystallized ligands in PDB complexes can be treated as frozen inverse shapes of their binding sites and can be used as templates to measure a compound’s binding probability to the corresponding targets through 3D similarity calculation. The more similarity between the compound and the crystallized ligand, the more likely the compound can form a similar shape and bind to the protein. Then, the similarity array between the compound and a pre-defined template set can be used as a virtual bioactivity profile (a multiple-dimensional molecular descriptor) in virtual screening.

On the other hand, proteins came into being over a long evolutionary process. The homologous or closely related proteins share similar sequences. As we all known, the number of druggable genes is in a limited number and the protein structures and functions are more conserved than their primary sequences [16]. Thus, these sequence-similar proteins tend to form a similar structure. Consequently, the protein or protein pocket (active sites) structural classes are also in a limited number. This conclusion can be demonstrated by the fact that there has been no new fold or superfamily submitted to the PDB in recent years [17,18,19,20,21]. In addition, long-term functional selection forced some proteins with dissimilar sequences to form similar active sites (the ligands induced the formation of enzyme/receptor structures). For example, the 5-HT_3A_ receptors are ion channels, while the other 5-HT receptors (5-HT_1,2,4–7_) are G protein-coupled receptors (GPCR) [22]. And, for the same reason, most drugs bind to more than one target, which is defined as drug promiscuity or polypharmacology. Therefore, we believe, the protein pocket classes are limited and can be represented with PDB structures.

In this article, based on these hypotheses discussed above: (1) the structural classes of protein pockets (accumulated in PDB) are limited in number; (2) the shape features of a pocket can be reflected by its ligands; (3) high similarity between a compound and the PDB ligand indicates possible binding with the corresponding target; we proposed a protocol to calculate the shape similarity profile based on PDB ligands and applied it in ligand-based virtual screening and QSAR study. We termed this method the Three-Dimensional Biologically Relevant Spectrum (BRS-3D) after our related 2D approach [23]. Firstly, we selected 300 diverse ligands from the sc-PDB to compose the 3D Biologically-relevant Representative Compound Database (BRCD-3D), which were used as templates for the BRS-3D calculation. Then, predictive discriminant models were established for 42 benchmark data sets using BRS-3D and the SVM algorithm. We compared the performance of BRS-3D and other state-of-the-art 2D and 3D molecular descriptors. We also applied the BRS-3D approach in histone deacetylase 1 (HDAC1) inhibitors screening and GPCR subtype selectivity prediction.

## 2. Results

### 2.1. Summary of BRCD-3D

Based on the self-similarity matrix and cluster analysis, a diverse set of ligands was extracted from the sc-PDB to compose the BRCD-3D. The size of the BRCD-3D is critical for the calculation efficiency and application effectiveness of BRS-3D. Therefore, we prepared a series of BRCD-3D databases with 500, 300, 200, 100, or 50 ligands. The prediction performances of BRCD-3D databases with different sizes were compared with two data sets from the ChEMBL [4]: 1189 human acetylcholinesterase (AChE) inhibitors and 1024 HIV-1 protease inhibitors. Two thousand random molecules selected from the Available Chemicals Directory (ACD) [24] were used as the negative samples. As shown in Supplementary Materials Figure S1, Tables S1 and S2, the *Accuracy*, *Precision*, *Recall*, and *MCC* values of the models decreased when the BRCD-3D size was reduced. The performance of the models with BRCD-3D size of 300 was close to the performance with a size of 500, while further reducing BRCD-3D size affected the discriminant efficiency. Thus, a BRCD-3D size of 300 was chosen to balance the computational consumption and modeling performance. Information regarding to the 300 ligands and their targets are provided in the Excel file in the Supplementary Materials. The ligand structures are also provided in a zipped file (mol2 format).

**Figure 1:**
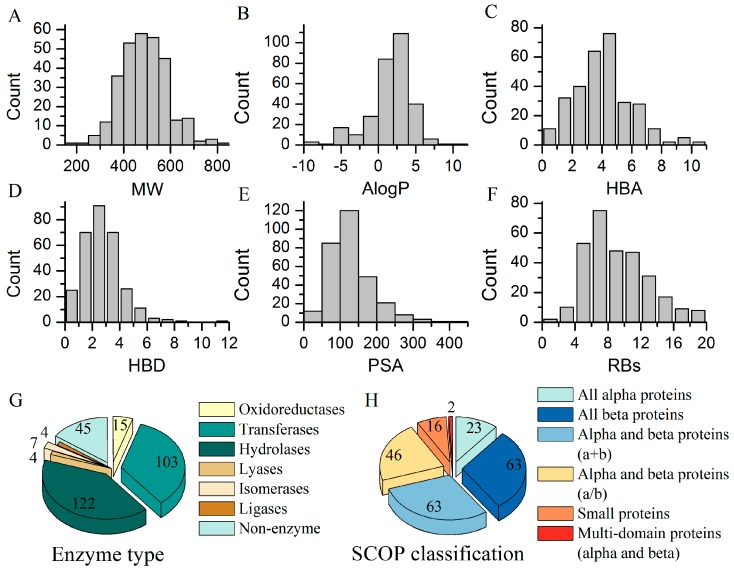
Physicochemical properties of BRCD-3D (3D Biologically-relevant Representative Compound Database) ligands and classifications of their corresponding targets. (**A**–**F**) Properties distribution of the ligands, calculated by Pipeline Pilot 8.5. MW: molecular weight; AlogP: the octanol-water partition coefficient; HBAs: the count of hydrogen bond acceptors; HBDs: the count of hydrogen bond donors; PSA: polar surface area; RBs: the number of rotatable bonds; (**G**) Pie chart of the enzyme types of the targets. Details are shown in Supplementary Materials Table S3; (**H**) Pie chart of the SCOP classification of the targets. Entries without SCOP annotations are not taken into account. Details are shown in Supplementary Materials Table S4.

The 300 ligands in the BRCD-3D included 281 putative ligands, 14 oligopeptides, and 5 cofactors. The peptides were composed of eight or fewer residues. We analyzed the physicochemical property distribution of the BRCD-3D ligands (Figure 1A–F), including molecular weight (MW), octanol-water partition coefficient (AlogP), number of hydrogen bond acceptors (HBAs), number of hydrogen bond donors (HBDs), polar surface area (PSA), and number of rotatable bonds (RBs). The MW of the ligands ranged from 140 to 800 Da. Most of the ligands conformed to Lipinski’s rule-of-five [25,26].

The biological diversity of the BRCD-3D ligands could be analyzed with their corresponding targets, as shown in the pie charts in Figure 1. The targets were categorized into seven classes: oxidoreductase, transferase, hydrolase, lyase, isomerase, ligase, and non-enzyme (the left pie chart, Figure 1G). Enzymes and GPCRs are the most important therapeutic targets in current drug discovery [27,28,29,30,31]. There were 255 enzymes, including 80 kinases and 90 proteases, representing the main part of BRCD-3D ligands’ targets. The structural classifications of the 300 targets were annotated according to SCOP (Structural Classification of Proteins, 1.75 release) [20] (the right pie chart, Figure 1H). Detailed information is presented in the Supplementary Materials Tables S3–S5.

### 2.2. Evaluation with GLL/GDD (G Protein-Coupled Receptor (GPCR) Ligand Library and the GPCR Decoy Database) Benchmark Data Sets

**Figure 2:**
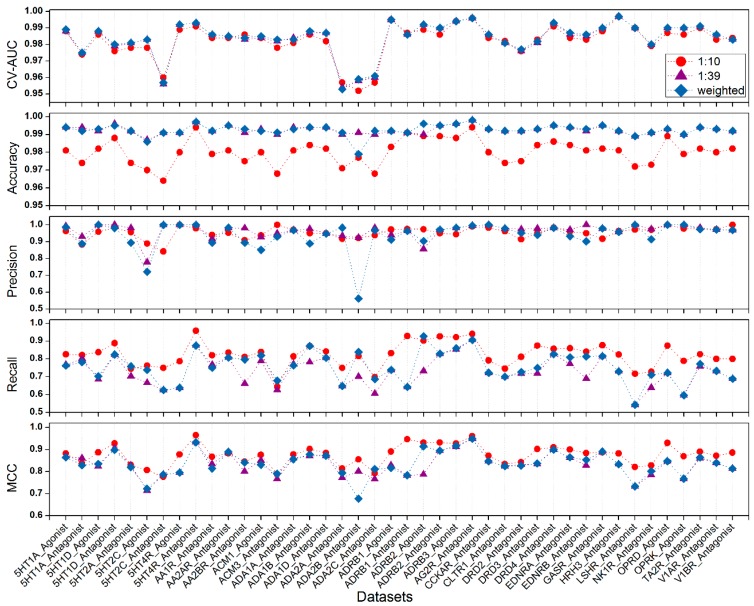
Comparison of the three methods in handling the data imbalance. The red circle denotes model results based on data sets with the proportion of 1:10 (ligands:decoys). The purple triangle denotes model results based on data sets with the original ratio (1:39). The blue diamond denotes results of models with different weight for ligands class and decoys class in the SVM model training.

Predictive discriminant models were successfully constructed using BRS-3D (Figure 2 and Supplementary Materials Table S6). We compared the performances of the three methods used to handle the unbalanced data. For the “1:10” data reduction method, the *Recall* values of most models were greater than the other two. However, reducing the number of decoy compounds caused information loss. The overall prediction accuracies (*ACC*) of the models were worse than the other methods, implicating that there were more false positives. For the “weighted” method (Table 1), the cross-validation AUC values were greater than 0.95 for all data sets, indicating that the SVM models had a fairly reliable learning ability. The *ACC* values for the test sets were greater than 0.95 for all models. The *Precision* values of most models were also acceptable. However, due to the data imbalance, the average *Recall* was approximately 0.76, meaning that the SVM models were biased towards predicting the compounds as decoys. The 1:39, 1:10, and weighted methods resulted in 40, 39, and 34 models with *Precision* values greater than 0.9, respectively. These models can effectively enrich the active compounds from decoys. The lowest *Precision* values for the 1:39, 1:10, and weighted methods were 0.788 (6th, 5HT2C_Agonist), 0.842 (7th, 5HT2C_Antagonist), and 0.562 (19th, ADA2B_Antagonist), respectively. These models could lead to high false positive rates. Except one data set (ADA2B_Antagonist), all the other *MCC* values were greater than 0.7, indicating that the models were acceptable.

The GLL/GDD was designed as a benchmark data set for a structure-based method (such as docking). Gatica et al. studied some targets in this data set with the docking approach. There were six common targets among their study and ours (Supplementary Materials Table S7). The maximum enrichment factors (EF_max_) ranged from 1.7 to 38.2, according to their docking approach [32]. In comparison, prediction based on BRS-3D models can reach EFs at 10–38 (top 10% and 2% in test sets). More importantly, our approach can be applied to systems with no known target structures.

In summary, the results of the SVM models based on benchmark data sets demonstrate that BRS-3D can effectively characterize the 3D structural features of molecules and can be used as a multi-dimensional structural descriptor. Combined with appropriate machine learning methods, prediction models can be developed to identify target-specific active compounds.

### 2.3. Feature Selection

**Figure 3:**
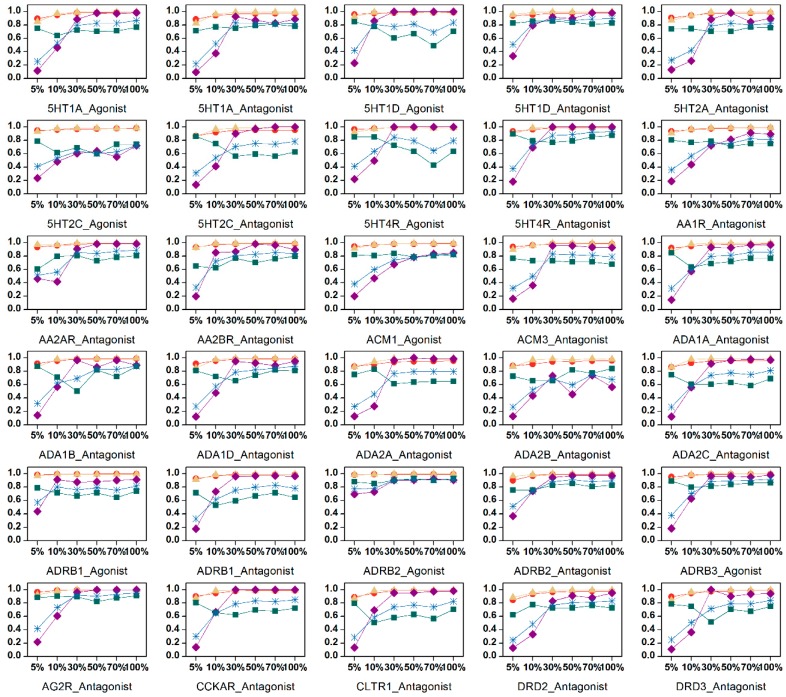

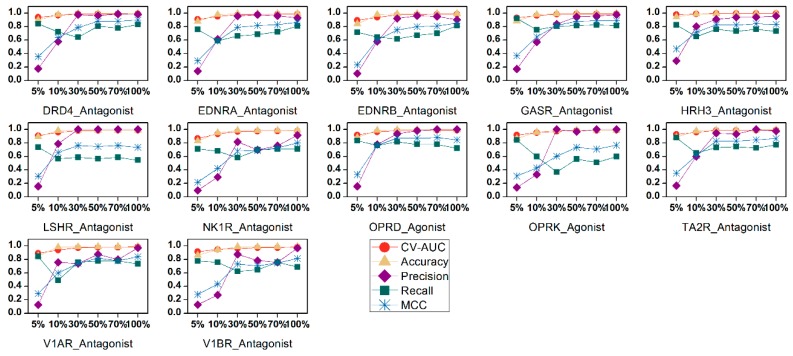
Comparison of SVM models using different BRS-3D feature subsets. For most data sets, the prediction performances were improved with the increasing of feature numbers.

We studied the influence of different feature subsets on the performance of the 42 SVM models. Weighted *C* parameter was used to handle the data imbalance. The results are presented in Figure 3. The cross-validation AUC values of models based on different feature subsets were acceptable (mostly over 0.9), again confirming the excellent learning ability of the method. For the test sets, the *ACC* values were mostly over 0.9. The influence of feature selection on these two statistical parameters was negligible. However, different feature subsets do affect the predictive ability, as shown by the variations of the *Precision*, *Recall*, and *MCC* values. The predictive ability was enhanced as more variables were added to the model. Most of the models with minimum feature numbers (5%) had the lowest *Precision*, meaning that they produced higher false positive results. When the feature number increased to 30% (90 BRS-3D variables), most models reached acceptable *Precision* and *MCC* values. After that, when more features were added, the trends became complex, meaning that the new added variables provided useful information for model construction but also brought some noises to the models. For most of the data sets, models with full BRS-3D performed best, as judged from the parameters. This behavior demonstrates the effectiveness of reducing the original 9878 sc-PDB ligands to 300 with cluster analysis during the BRCD-3D construction process. Of course, the models should be analyzed with more sophisticated statistical parameters for real screening, such as ROCFIT and ROCED [33], which exceed the scope of this article.

### 2.4. Comparison with Other Molecular Descriptors

**Figure 4:**
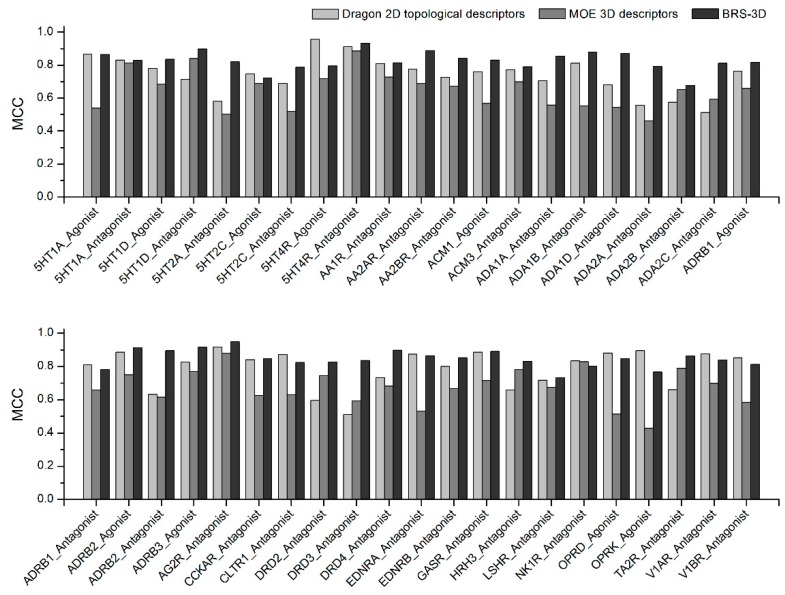
The *MCC* values of SVM models based on BRS-3D and other two state-of-the-art descriptors.

The prediction performances of BRS-3D-based models were compared with models based on Dragon 2D and MOE 3D descriptors. The results are shown in Table 2. The prediction *Accuracy* values of all the three descriptors are sufficiently high, but the *Precision* values of BRS-3D are much higher than Dragon 2D descriptors and MOE 3D descriptors, which means the BRS-3D based models have the lowest false positive rates. Although the *Recall* values of BRS-3D are slightly lower than the other two descriptors, the models based on BRS-3D still show sufficient sensitivity. We compared the *MCC* values of these three descriptors (Figure 4), which are considered as a comprehensive evaluation index for classification models. For 29 of all the 42 data sets, the BRS-3D-based models possessed the highest *MCC* values. For the other 13 data sets, BRS-3D performed as well as Dragon 2D topological descriptors, and both these two descriptors performed better than MOE 3D descriptors.

### 2.5. HDAC1 Inhibitor Screening

HDAC1 is an attractive drug target for cancer therapy [34]. Vorinostat (SAHA), the inhibitor of HDAC1, has been approved by the FDA as an effective drug for the treatment of cutaneous T cell lymphoma [35]. Therefore, we applied BRS-3D in the screening for HDAC1 inhibitors. The candidate database for virtual screening contained more than 300,000 drug-like or lead-like compounds derived from Specs, ChemDiv, and Enamine. We used only two screening filters, the pharmacophore model (refer to Supplementary Materials Figure S2) and the BRS-3D discriminant model (refer to Supplementary Materials Table S8), to simplify the study. At last, 30 molecules were selected and purchased. The activity assay results showed that two similar molecules could inhibit HDAC1 at micromolar concentrations. The inhibition rates of these two molecules were 34.65% and 38.66% at 10 μM. The IC_50_ values calculated by curve fitting were 43.99 and 30.07 μM, respectively (Supplementary Materials Figure S3).

### 2.6. Application of BRS-3D in Subtype Selectivity Predictions

We noticed that one shortcoming of molecular superimposing was that shape played the most important role in superimposition scoring, while fewer pharmacophore features were taken into consideration. This limited the application of BRS-3D in activity prediction for targets that were charge- or H-bond-sensitive. Different subtypes of a GPCR family can be activated by the same ligand. We believed that the subtype selectivity of GPCR ligands was dominated largely by dynamic conformation-changing patterns. For example, all dopamine receptors (DR_1–4_) can be activated by dopamine or its mimics [36]. The pharmacophore distributions of the ligands are similar to each other. The selectivity of DR ligands depends on their dynamic shape or conformation-changing patterns. Because BRS-3D is calculated with multiple superimposing templates, it can encode information about multiple-conformation and then can be applied to GPCR subtype selectivity prediction. We applied BRS-3D in subtype selectivity prediction of dopamine receptors (DR) [37], adenosine receptors (AR) [38], cannabinoid receptors (CB), and an enzyme, monoamine oxidase (MAO). Predictive models could be constructed for these systems. In this paper, we report the result of CB subtype selectivity prediction as an example.

Cannabinoid receptors include two subtypes, denoted as CB_1_ and CB_2_. CB_1_ selective antagonists show clinical efficacy in the treatment of obesity, metabolic disorders, and drug abuse [39,40], whereas CB_2_ selective agents demonstrate efficacy in inflammatory pain models [41] and play neuroprotective roles in Huntington’s and Alzheimer’s diseases [42,43]. The development of subtype-selective compounds for CB_1_/CB_2_ receptors can not only avoid the unwanted side effects produced by nonselective ligands, but also help us understand the specific physiological functions of each subtype receptor.

**Figure 5:**
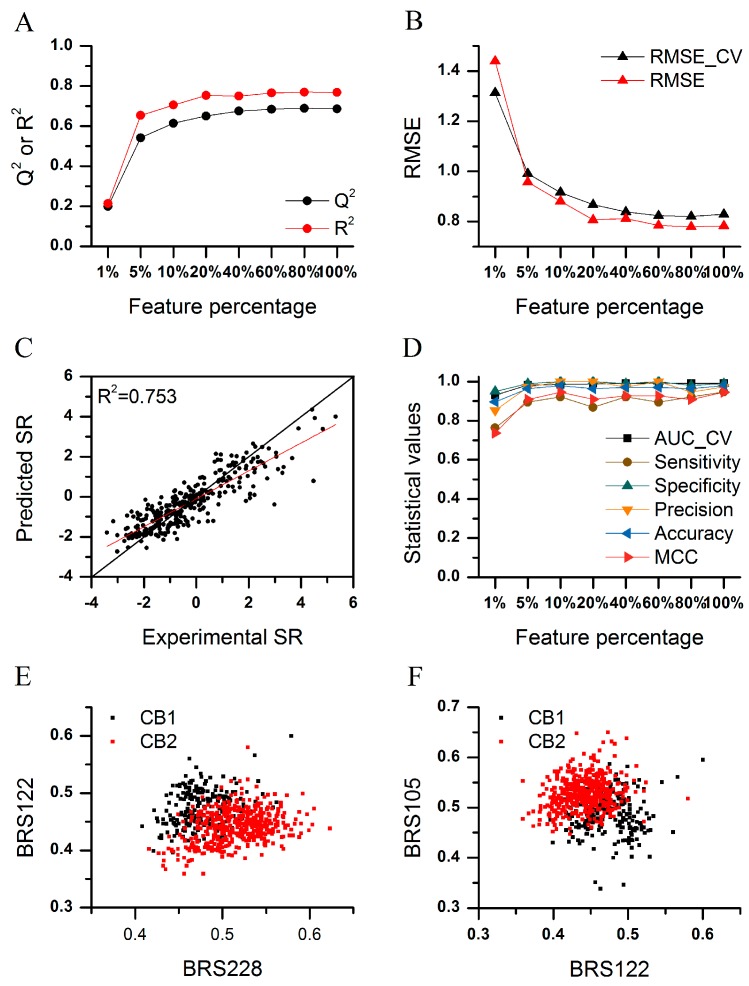
The CB_1_/CB_2_ subtype selectivity prediction models. (**A**) Cross-validation *Q*^2^ and test set *R*^2^ of the regression models with different feature subsets; (**B**) cross-validation and test set RMSE of the regression models with different feature subsets; (**C**) relationship between experimental and predicted SR of the model with 20% BRS-3D features; (**D**) discriminant models with different feature subsets; and (**E**,**F**) distribution of the selective compounds in the chemical space, composed of the most important features.

We extracted all the compounds with definite Ki values for both CB_1_ and CB_2_ receptors (human sapiens) in ChEMBL (version 20). The selectivity ratio (SR) was defined as SR_CB1/CB2_ = pKi_CB1_ − pKi_CB2_. The compounds with SR ≥ 1.3 or SR ≤ −1.3 were considered as CB_1_/CB_2_ selective ones. As shown in Figure 5 and Tables S9 and S10, prediction regression and discriminant models were successfully constructed. The performances of the models improved with increasing of used features in the model development. The regression model reached satisfactory performance with the cross-validated *Q*^2^ = 0.650 for the training set and *R*^2^ = 0.753 for the test set (Figure 5A), when 20% features (60 variables) were employed. The RMSE of the training set and test set were lower than 1 unit (Figure 5B). Considering that the compounds in ChEMBL were collected from different laboratories, the results were excellent. We conducted a data resampling (100 times, Figure S4A,B) and Y-randomization test (500 times, Figure S4C) to evaluate the stability of the prediction models and to exclude possible chance correlation. We also analyzed the applicability domain of the model (Figure S4D). The discriminant models were simpler than regression models, since only 10% features (30 variables) were needed (Figure 5D) when over 90% of selective ligands could be distinguished correctly. The prediction accuracy (0.896) was still satisfactory, with only 3 BRS-3D features (1%).

We analyzed the distribution of the selective compounds in the chemical space composed of the most important BRS-3D features (Figure 5E,F). As the results show, the CB_1_-selective and CB_2_-selective compounds distributed in different zones in the space. The compounds with higher similarity to the 105th and 228th BRCD-3D ligands and with lower similarity to the 122th BRCD-3D ligand were biased to bind to the CB_2_ receptor, and vice versa. We calculated the similarity of CB_1_/CB_2_- selective compounds with active compounds in ChEMBL of the corresponding targets of BRS228, BRS122 and BRS105 (with PDB ID 2WIH, 1QBR and 1R1H, respectively). Surprisingly, the similarities of CB_1_- and CB_2_-selective compounds with the active compounds had no difference (BRS122 and BRS105) or weak inversed trends (BRS128). The results demonstrated the advantages of 3D approaches relative to 2D ones. That is, activity relationships related to molecular shapes could be discovered with 3D methods, even when the topological structures of the compounds were dissimilar to each other. Figure S5 gives examples of the five most selective compounds of CB_1_ and CB_2_. Thus, 3D methods were more suitable for scaffold hopping [12].

## 3. Discussion

In this paper, we introduced a multi-dimensional molecular descriptor, BRS-3D. The compounds under scrutiny were superimposed on a diverse set of 300 ligands selected from the sc-PDB. Then, the shape similarity profile was used as a multi-dimensional descriptor for QSAR studies. Predictive SVM models were successfully constructed to discriminate active molecules from decoys, and most of the models performed well. Comparison with two other state-of-the-art molecular descriptors showed that the models based on BRS-3D achieved the best prediction performance. We also applied this approach in a real screening project for HDAC1 inhibitors. Two of the 30 compounds showed moderate activity, with IC_50_ values of 30.07 and 43.99 μM. Predictive regression models and discriminant models were constructed for CB_1_/CB_2_ subtype selectivity prediction. Therefore, we believe that BRS-3D is a valid molecular descriptor for ligand-based virtual screening and QSAR studies.

Recently, ligand profiling, such as the Cerep BioPrint^®^ profile and Novartis HTSFPs high-throughput screening fingerprints), gained much attention [44,45,46,47,48,49]. Helal and co-workers used the PubChem Bioassay database to build a publicly available version of HTSFPs [50]. In these works, a compound was encoded with its biological activities profile, which was collected from a battery of in vitro pharmacology or ADME assays. Gene-expression profile (C-map) was also used as a molecular descriptor to study the relationships between small molecules, genes, and diseases [51]. All of these works demonstrated that the biological activity, or similar profile approaches, were efficient for virtual screening and target prediction. In fact, ligand profiling can also be performed with theoretical calculation, e.g., inverse docking [52] and pharmacophore database mapping [53]. In 2012, Sato et al. proposed a shape overlay similarity profile with known active compounds and used it as molecular descriptors in machine learning for ligand-based virtual screening [54]. Taking known active compounds as templates, they calculated the 3D similarity profile (the array of overlay scoring) and used these profiles as explanatory variables. Predictive discriminant models were constructed using the support vector machine (SVM). No active conformations are needed during this process. When diverse active compounds are available, this protocol can overcome the shortcomings of traditional 3D methods. That is to say, without strict substructure alignment or active conformation, the screening protocol can be processed automatically. However, using active compounds of a specific target as superimposition templates made this descriptor not reusable. When the target changes, the templates must be renovated and the similarity array has to be calculated again.

Similar to Sato’s protocol, our approach is also a theoretically implement of activity profiling. Nevertheless, there are two differences between these two approaches. Firstly, we used a fixed set of templates to make the calculated descriptor reusable for new systems. Secondly, using a diverse set of irrelevant ligands as references attach more biological significance to the BRS-3D scores. The high similarity means that the objective compound can form a similar shape with the corresponding ligand and can bind to the corresponding target, while the dissimilar (with a low superimposing score) indicates that the compound under scrutiny cannot form similar conformations with the PDB ligands (forbidden conformations), which may cause possible confliction with the target or shape mismatching.

In fact, the fitting profile of an objective molecule against the 300 targets in the BRCD-3D can also be calculated by reverse docking. We compared the performances of docking-based BRS-3D and superimposing-based BRS-3D using the data set of 1189 AChE inhibitors and 2000 diverse compounds from the ACD database. Surflex-Sim outperformed Surflex-Dock (Supplementary Materials Table S11), possibly due to the poor scoring functions of current docking programs.

BRS-3D is a ligand-based method. Therefore, it can be used for systems without crystallized target structures. As an example, GPCRs are the targets of over 30% of marketed prescription drugs [55]. Only a few crystal structures of GPCRs have been resolved, limiting the application of structure-based methods. In this paper, the results show that BRS-3D can be applied to GLL/GDD discrimination. BRS-3D is calculated with multiple templates. Therefore, BRS-3D reflects the conformational ensemble (300 possible binding modes), which is useful for modeling of the dynamic binding process between the objective compound and its potential targets. Also, for the same reason, BRS-3D may also be applied in drug discovery for multi-target projects. Different from conventional 3D-QSAR methods, such as CoMFA and CoMSIA, BRS-3D belongs to the second type of QSAR method discussed by Fujita and Winkler [56]. When the BRS-3D model was constructed and validated, it could be used in preliminary virtual screening to identify new scaffolds automatically.

In addition to the advantages discussed above, BRS-3D also has some drawbacks. First, shape similarity calculation is highly computationally sensitive: it takes approximately 30 min to calculate the BRS-3D for a typical molecule on a modern CPU core (we used the Intel Xeon E5-2609 v2 @ 2.50 GHz). Therefore, this method is not suitable for on-the-fly analysis. Nevertheless, the BRS-3D descriptor only needs to be calculated once, and the results can then be reused in different projects. Currently, we have finished the calculations for more than 800,000 drug-like compounds. The calculated profiles have been stored in an in-house database for further usage. Second, although each BRS-3D element has a definite meaning, i.e., the similarity to a BRCD-3D ligand, models developed using this descriptor and machine learning methods were less amenable to interpretation. Therefore, it is difficult to draw a rule and to guide the rational design of new active compounds. However, as illustrated in Figure 5E,F, the distributions of the compounds in a BRS-3D space can provide valuable information for inferring the relationships between objective compounds and BRCD-3D targets, which is useful in lead optimization and also in drug repositioning [57]. Third, we used Surflex-Sim for shape similarity calculation. However, the similarity scores calculated with this method have a centralized distribution, i.e., most similarity scores ranged from 0.3 to 0.7. BRS-3D can characterize the surface and shape properties of the molecules under study. As the shape similarity cannot encode electronic and other polar features, we combined the pharmacophore method with our method for screening HDAC1 inhibitors (which also enhances the screening speed).

In summary, BRS-3D can be used as a multi-dimensional molecular descriptor in ligand-based studies. Calculated using multiple templates, BRS-3D can reflect the transformation pattern between the active and inactive conformations of the molecule under scrutiny. Of course, as required for all 3D methods, the active compounds should bind to the same pocket of the target in a similar manner.

## 4. Materials and Methods

### 4.1. Workflow of BRS-3D-Based Virtual Screening

**Scheme 1:**
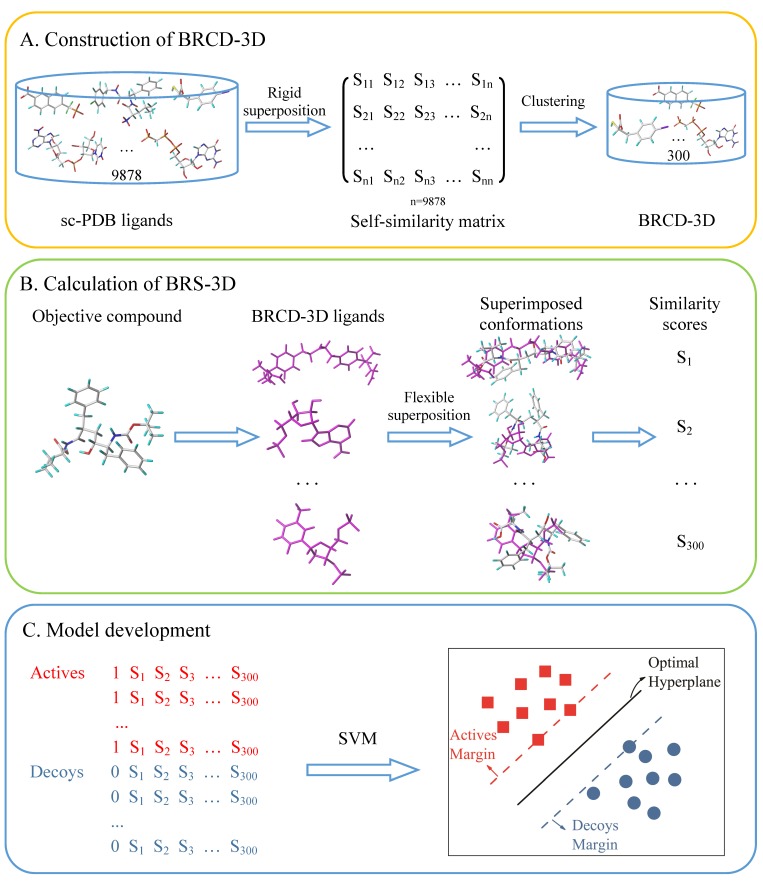
Workflow of QSAR study based on BRS-3D. The process contains three steps: (**A**) Construction of BRCD-3D. 3D shape similarity calculations and structural clustering were used to extract a set of 300 diverse ligands from the druggable protein-ligand database, sc-PDB. This ligand set was named the BRCD-3D; (**B**) Calculation of BRS-3D. The objective compound was flexibly superimposed onto the 300 BRCD-3D ligands (magenta ones), resulting in 300 similarity scores. The array of the scores was defined as BRS-3D, which could be used as a multi-dimensional molecular descriptor in virtual screening and QSAR studies; (**C**) Model development. Discriminant or regression models were developed with the machine learning methods (e.g., SVM), taking BRS-3D as the independent variable.

This study was divided into three steps: templates preparation, BRS-3D calculation, and model development and validation (Scheme 1). First, 3D shape similarity calculations and structural clustering were used to extract a set of 300 diverse ligands from the druggable protein–ligand complex database, sc-PDB, which was a subset of the original PDB [58]. This ligand set was named the BRCD-3D. Then, each of the molecules under scrutiny was flexibly superimposed onto the 300 ligands in the BRCD-3D. These superimpositions were scored according to the degree of shape overlap and property similarity, producing a 300 dimensional similarity array called the BRS-3D. Finally, quantitative or discriminant models were developed using the BRS-3D and various machine learning methods.

### 4.2. Surflex-Sim Superimposition

A variety of chemoinformatics tools are available for superimposition, such as FLEXS [59] and ROCS [60]. In this paper, we used Surflex-Sim for BRCD-3D construction and BRS-3D calculation. Surflex-Sim is the molecular similarity computing module of Surflex [61]. It measures the 3D similarity between two molecules based on the morphological similarity algorithm, which takes into account both the surface shape match and the similarity of charge characteristics [62]. The superimposing process can be divided into four steps, including fragmentation, conformational search, alignment, and scoring, which will be performed automatically by the Surflex-Sim program. More details about the superimposition method can be found in Jain’s paper [62]. Surflex-Sim similarity scores range from 0 to 1. A score greater than 0.7 is generally considered to indicate a significant functional relationship between molecules [62]. Default parameters were used for all of the calculations.

### 4.3. Construction of the BRCD-3D

The BRCD-3D is a representative collection of the active conformations of ligands. We used ligands in the sc-PDB to build the BRCD-3D. The sc-PDB is a ligand-target complex database derived from the PDB [58]. As this database was developed for drug discovery, only druggable binding sites and their corresponding ligands were included in the sc-PDB. Therefore, the sc-PDB could be used to represent the known bioactive conformational space. However, some ligands were co-crystallized in more than one PDB entry, such as most cofactors. And, the ligands that bound to the same pocket were highly similar. These redundancies should be removed.

We extracted all 9878 ligands from the sc-PDB (version 2011) [63]. A self-similarity matrix of the 9878 ligands was calculated through an iterative process of rigid molecular superimposition. In each iteration, a sc-PDB ligand was used as the template and kept rigid. All other ligands were also kept rigid and superimposed onto the template. The superimpositions were scored with the default Surflex-Sim parameters. These pairwise similarity scores constituted the self-similarity matrix.

Then, based on the similarity matrix and cluster analysis, a diverse subset of the 9878 ligands was extracted to compile the BRCD-3D. The 9878 ligands were clustered into several groups via an in-house protocol utilizing the component “Cluster Data” in Pipeline Pilot 8.5 [64]. In the clustering protocol, a row of the self-similarity matrix was used as a numeric descriptor to compute the distance between two ligands, and the distance function was set to “One Minus Pearson correlation”. Clustering was performed using the maximum dissimilarity method. The cluster centers were output as members of the BRCD-3D. We built five versions of the BRCD-3D database with different numbers of ligands (500, 300, 200, 100, 50) to compare their performances.

### 4.4. Calculation of BRS-3D

BRS-3D was defined as the shape similarity profile between the molecule under scrutiny and all of the ligands in the BRCD-3D. As the BRCD-3D consisted of a diverse range of ligands, the BRS-3D could serve as a GPS-like location system within the bioactive-conformation chemical space. To calculate the BRS-3D, the molecules under scrutiny (objective molecules) were flexibly superimposed onto BRCD-3D ligands that were kept rigid. By default, 10 overlapped conformations and similarity scores between the objective molecule and a template were output. Only the highest score was kept as one element of the BRS-3D. Therefore, the dimension of the BRS-3D was equal to the number of ligands in the BRCD-3D. BRS-3D was used as a multi-dimensional descriptor for the development of QSAR models.

### 4.5. The Benchmark Data Sets

We used the G protein-coupled receptor (GPCR) ligand library and the GPCR decoy database (GLL/GDD) to evaluate the efficiency of BRS-3D for ligand-based studies. GLL/GDD were compiled by the Cavasotto Laboratory [32], including active ligands (agonist and antagonist) of 147 human Class A rhodopsin-like GPCR targets and corresponding decoys. For each GLL ligand, there were 39 decoys. These decoys were selected from the ZINC database, with similar physicochemical properties (molecular weight, formal charge, hydrogen bond donors and acceptors, rotatable bonds, and logP) but dissimilar structure to the corresponding GLL ligand. GLL/GDD was originally developed for docking approaches, but also used for performance evaluation of ligand-based methods [65].

To evaluate the efficiency of our approach, SVM discriminant models were constructed for 42 GLL/GDD data sets with more than 200 ligands. The structures of the GLL/GDD data sets were downloaded from the website of the Cavasotto Laboratory [66]. Each data set was randomly divided into a training set and a test set at a ratio of 4:1. The training set was used to select the optimal parameter settings by cross-validation and to build prediction models. The test set was used only for model evaluation.

The ligands and decoys of the GLL/GDD data sets were unbalanced, with a ligand:decoys ratio of 1:39. We compared three methods of handling this unbalance. First, the original data were used without any special treatment (1:39 method). Second, we reduced the ligand:decoy ratio from 1:39 to 1:10 (1:10 method). For each ligand, only 10 of the 39 original decoys were randomly selected and used for model development, and the other decoys were discarded. In the third approach, we assigned different weight factors on parameter *C* (39 and 1 for the ligands and decoys, respectively) in the SVM models development (weighted method). The 42 data sets are summarized in Table 3.

### 4.6. Model Development and Validation

SVM is a promising machine learning method and has been extensively applied in various pattern recognition systems and across all fields of informatics, including bioinformatics and chemoinformatics [54,67,68,69,70,71]. Combined with molecular fingerprints or descriptors, SVM can be easily utilized for virtual screening with good prediction performance. In this study, LIBSVM (v3.16) [72], an implementation of SVM for classification, regression, and distribution estimation, was adopted to develop the discriminant models based on BRS-3D. GLL active compounds (agonists or antagonists) and GDD decoys were assigned as positive and negative samples, respectively. The RBF (radial basis function) was used as the kernel function, and 80% of the data set was used as the training set, while the rest was used as the test set. We used 10-fold cross-validation to verify the learning ability of the models. The parameter gamma of the kernel function and parameter *C* were optimized with grid searching. For the classification models, the area under the receiver operating characteristic curve (AUC) was used to evaluate the cross-validation results and to determine the best parameter settings. The parameter settings are generally considered to be acceptable when the cross-validation AUC value of a classifier is greater than 0.9. For the regression models, the cross-validation root-mean-square error (RMSE_CV) was used for parameter optimization.

In addition to cross-validation, we also verified the performance of the models with test sets (20% of the original data set). Statistical parameters including *Accuracy* (*ACC*), *Precision*, *Recall*, and Matthew’s correlation coefficient (*MCC*) were computed to assess the performance of the model.

(1) $$ACC=\frac{TP+TN}{TP+TN+FP+FN}$$

(2) $$Precision=\frac{TP}{TP+FP}$$

(3) $$Recall=Sensitivity=\frac{TP}{TP+FN}$$

(4) $$Specificity=\frac{TN}{TN+FP}$$

(5) $$MCC=\frac{TP\times TN-FP\times FN}{\sqrt{\left(TP+FP)(TP+FN)(TN+FP)(TN+FN\right)}}$$

Here, *TP*, *TN*, *FP*, and *FN* denote the number of true positives, true negatives, false positives, and false negatives, respectively. *ACC* is the overall prediction accuracy of a classifier. Higher *ACC* values indicate higher predictive power. However, if the data points of the two classes are highly unbalanced, *ACC* cannot correctly reflect predictive power. In this situation, *MCC* is preferred. *MCC* ranges from −1 to 1. *MCC* = 1 indicates an ideal prediction, while *MCC* = 0 represents a random prediction.

The RMSE, squared correlation coefficients of cross-validation (*Q*^2^) and the determination coefficient (*R*^2^) of test sets were calculated for regression models.

(6) $$RMSE=\sqrt{\frac{1}{n}\sum {\left(y-\hat{y}\right)}^{2}}$$

(7) $$Q^{2}={\left(\frac{\sum \left(y-\bar{y})(\hat{y}-\bar{\hat{y}}\right)}{\sqrt{{\sum {\left(y-\bar{y}\right)}^{2}\sum \left(\hat{y}-\bar{\hat{y}}\right)}^{2}}}\right)}^{2}$$

(8) $$R^{2}=1-\frac{\sum {\left(y-\hat{y}\right)}^{2}}{\sum {\left(y-\bar{y}\right)}^{2}}$$

Here, n is the number of samples, y is the observed response variable, $\hat{y}$ is the corresponding predicted value, $\bar{y}$ and $\bar{\hat{y}}$ are the mean values of y and $\hat{y}$, respectively. As recommended by Alexander et al. [73], *R*^2^ > 0.6 and low RMSE for the test set indicate the satisfied prediction ability of the regression models.

### 4.7. Feature Selection

To assess the influence of feature size on SVM model performance, several different feature subsets of BRS-3D were selected to build the prediction models. A total of 42 Random Forest (RF) models were built, using BRS-3D as variables. This process was implemented by the component “Learn R Forest Model” in Pipeline Pilot 8.5. First, the importance of each variable in the BRS-3D was measured with RF models, using the method of permutation accuracy importance [74]. Then, feature subsets with 15 (top 5%), 30 (top 10%), 90 (top 30%), 150 (top 50%), and 210 (top 70%) variables were selected according to the ranked importance of the variables. The feature subsets were used to build LIBSVM discriminant models. The feature selection process was performed with the original 1:39 unbalanced data sets. The SVM model performances were compared with and without feature selection.

### 4.8. Dragon 2D Descriptors and MOE 3D Descriptors

Two kinds of state-of-the-art molecular descriptors were adopted to build SVM discriminant models for the same 42 benchmark data sets. Their prediction performances were compared with BRS-3D. Totally, 107 topological (2D) descriptors were calculated with Dragon (version 5.4) [75]; 91 surface area-, volume-, and shape-related 3D descriptors were also calculated, using MOE 2009 [76]. The details of Dragon 2D and MOE 3D descriptors are listed in the Supplementary Materials, Tables S12 and S13. These two kinds of molecular descriptors were used as explanatory variables to build SVM models. The performance (*ACC*, *Precision*, *Recall*, and *MCC*) of Dragon 2D-, MOE 3D-, and BRS-3D-based models were compared.

## Figures and Tables

**Table 1:** Results of the discriminant models for the 42 GLL/GDD (G protein-coupled receptor (GPCR) ligand library and the GPCR decoy database) data sets. Models were built with the “weighted” method for handling the data imbalance. The CV AUC is a 10-fold cross-validation result of the training set. *Accuracy*, *Precision*, *Recall*, and *MCC* are the prediction results for the test set. Results of the other two treatments for data imbalance can be found in Supplementary Materials Table S6.

No.	Data Sets	CV AUC	Accuray	Precision	Recall	MCC
1	5HT1A_Agonist	0.989	0.994	0.986	0.763	0.865
2	5HT1A_Antagonist	0.975	0.992	0.888	0.782	0.829
3	5HT1D_Agonist	0.988	0.993	1.000	0.703	0.835
4	5HT1D_Antagonist	0.980	0.995	0.981	0.825	0.898
5	5HT2A_Antagonist	0.981	0.992	0.894	0.759	0.820
6	5HT2C_Agonist	0.983	0.986	0.721	0.738	0.722
7	5HT2C_Antagonist	0.957	0.991	1.000	0.625	0.787
8	5HT4R_Agonist	0.992	0.991	1.000	0.638	0.795
9	5HT4R_Antagonist	0.993	0.997	1.000	0.875	0.933
10	AA1R_Antagonist	0.986	0.992	0.894	0.750	0.814
11	AA2AR_Antagonist	0.985	0.995	0.983	0.808	0.889
12	AA2BR_Antagonist	0.984	0.993	0.894	0.797	0.841
13	ACM1_Agonist	0.985	0.992	0.851	0.820	0.831
14	ACM3_Antagonist	0.983	0.991	0.930	0.678	0.790
15	ADA1A_Antagonist	0.983	0.993	0.968	0.763	0.856
16	ADA1B_Antagonist	0.988	0.994	0.889	0.873	0.878
17	ADA1D_Antagonist	0.987	0.994	0.948	0.807	0.872
18	ADA2A_Antagonist	0.953	0.991	0.983	0.648	0.794
19	ADA2B_Antagonist	0.959	0.979	0.562	0.839	0.677
20	ADA2C_Antagonist	0.961	0.992	0.967	0.686	0.811
21	ADRB1_Agonist	0.995	0.992	0.912	0.738	0.816
22	ADRB1_Antagonist	0.986	0.991	0.964	0.643	0.783
23	ADRB2_Agonist	0.992	0.996	0.904	0.927	0.914
24	ADRB2_Antagonist	0.990	0.995	0.971	0.829	0.895
25	ADRB3_Agonist	0.994	0.996	0.982	0.860	0.917
26	AG2R_Antagonist	0.996	0.998	0.996	0.907	0.949
27	CCKAR_Antagonist	0.986	0.993	1.000	0.722	0.847
28	CLTR1_Antagonist	0.981	0.992	0.979	0.701	0.825
29	DRD2_Antagonist	0.977	0.992	0.951	0.726	0.827
30	DRD3_Antagonist	0.982	0.993	0.941	0.750	0.837
31	DRD4_Antagonist	0.993	0.995	0.982	0.827	0.899
32	EDNRA_Antagonist	0.987	0.994	0.932	0.809	0.865
33	EDNRB_Antagonist	0.986	0.993	0.902	0.814	0.853
34	GASR_Antagonist	0.990	0.995	0.979	0.816	0.891
35	HRH3_Antagonist	0.997	0.992	0.958	0.730	0.833
36	LSHR_Antagonist	0.990	0.989	1.000	0.543	0.733
37	NK1R_Antagonist	0.980	0.991	0.914	0.711	0.802
38	OPRD_Agonist	0.990	0.993	1.000	0.722	0.847
39	OPRK_Agonist	0.990	0.990	1.000	0.596	0.768
40	TA2R_Antagonist	0.991	0.994	0.974	0.772	0.864
41	V1AR_Antagonist	0.986	0.993	0.971	0.733	0.840
42	V1BR_Antagonist	0.983	0.992	0.969	0.689	0.813

**Table 2:** SVM models based on Dragon 2D, MOE 3D, and BRS-3D descriptors.

Data Sets	Accuracy			Precision			Recall			MCC		
	Dragon 2D	MOE 3D	BRS-3D	Dragon 2D	MOE 3D	BRS-3D	Dragon 2D	MOE 3D	BRS-3D	Dragon 2D	MOE 3D	BRS-3D
1	0.993	0.951	0.994	0.849	0.330	0.986	0.889	0.932	0.763	0.866	0.539	0.865
2	0.992	0.991	0.992	0.819	0.814	0.888	0.851	0.822	0.782	0.831	0.813	0.829
3	0.987	0.977	0.993	0.671	0.526	1.000	0.919	0.919	0.703	0.779	0.686	0.835
4	0.981	0.992	0.995	0.568	0.831	0.981	0.921	0.857	0.825	0.715	0.840	0.898
5	0.964	0.945	0.992	0.401	0.300	0.894	0.883	0.903	0.759	0.581	0.502	0.820
6	0.987	0.980	0.986	0.744	0.571	0.721	0.762	0.857	0.738	0.747	0.691	0.722
7	0.983	0.949	0.991	0.636	0.317	1.000	0.766	0.906	0.625	0.690	0.519	0.787
8	0.998	0.979	0.991	0.939	0.541	1.000	0.979	0.979	0.638	0.957	0.719	0.795
9	0.995	0.994	0.997	0.855	0.863	1.000	0.979	0.917	0.875	0.912	0.886	0.933
10	0.990	0.986	0.992	0.774	0.705	0.894	0.857	0.769	0.750	0.810	0.729	0.814
11	0.989	0.982	0.995	0.756	0.602	0.983	0.808	0.808	0.808	0.776	0.689	0.889
12	0.987	0.978	0.993	0.794	0.539	0.894	0.676	0.865	0.797	0.726	0.672	0.841
13	0.986	0.962	0.992	0.662	0.383	0.851	0.888	0.882	0.820	0.760	0.567	0.831
14	0.988	0.982	0.991	0.714	0.605	0.930	0.847	0.831	0.678	0.772	0.700	0.790
15	0.980	0.960	0.993	0.557	0.373	0.968	0.915	0.881	0.763	0.705	0.558	0.856
16	0.990	0.955	0.994	0.758	0.349	0.889	0.882	0.927	0.873	0.812	0.554	0.878
17	0.977	0.955	0.994	0.528	0.347	0.948	0.904	0.904	0.807	0.681	0.544	0.872
18	0.957	0.938	0.991	0.359	0.270	0.983	0.909	0.864	0.648	0.556	0.462	0.794
19	0.973	0.982	0.979	0.471	0.632	0.562	0.736	0.690	0.839	0.576	0.651	0.677
20	0.955	0.978	0.992	0.335	0.542	0.967	0.837	0.674	0.686	0.513	0.593	0.811
21	0.986	0.974	0.992	0.655	0.494	0.912	0.905	0.905	0.738	0.763	0.658	0.816
22	0.989	0.977	0.991	0.702	0.522	0.964	0.952	0.857	0.643	0.812	0.659	0.783
23	0.995	0.987	0.996	0.900	0.694	0.904	0.878	0.829	0.927	0.886	0.752	0.914
24	0.974	0.971	0.995	0.452	0.429	0.971	0.917	0.917	0.829	0.633	0.616	0.895
25	0.990	0.986	0.996	0.738	0.665	0.982	0.938	0.907	0.860	0.827	0.770	0.917
26	0.996	0.994	0.998	0.877	0.843	0.996	0.967	0.927	0.907	0.918	0.881	0.949
27	0.992	0.972	0.993	0.857	0.467	1.000	0.833	0.875	0.722	0.841	0.627	0.847
28	0.994	0.968	0.992	0.857	0.441	0.979	0.896	0.940	0.701	0.873	0.632	0.825
29	0.964	0.987	0.992	0.403	0.699	0.951	0.925	0.811	0.726	0.597	0.746	0.827
30	0.948	0.975	0.993	0.313	0.500	0.941	0.891	0.734	0.750	0.510	0.594	0.837
31	0.981	0.975	0.995	0.575	0.502	0.982	0.955	0.955	0.827	0.733	0.683	0.899
32	0.994	0.951	0.994	0.864	0.329	0.932	0.890	0.912	0.809	0.874	0.531	0.865
33	0.990	0.975	0.993	0.758	0.505	0.902	0.858	0.912	0.814	0.801	0.668	0.853
34	0.994	0.981	0.995	0.861	0.582	0.979	0.921	0.904	0.816	0.887	0.717	0.891
35	0.977	0.988	0.992	0.524	0.736	0.958	0.857	0.841	0.730	0.660	0.781	0.833
36	0.986	0.985	0.989	0.733	0.744	1.000	0.717	0.630	0.543	0.718	0.677	0.733
37	0.992	0.992	0.991	0.805	0.830	0.914	0.872	0.839	0.711	0.834	0.830	0.802
38	0.994	0.946	0.993	0.867	0.307	1.000	0.903	0.917	0.722	0.882	0.513	0.847
39	0.995	0.935	0.990	0.869	0.251	1.000	0.930	0.807	0.596	0.896	0.428	0.768
40	0.973	0.988	0.994	0.479	0.721	0.974	0.945	0.876	0.772	0.662	0.789	0.864
41	0.994	0.987	0.993	0.870	0.800	0.971	0.889	0.622	0.733	0.876	0.699	0.840
42	0.992	0.969	0.992	0.768	0.435	0.969	0.956	0.822	0.689	0.853	0.585	0.813

**Table 3:** The 42 GLL/GDD data sets with more than 200 ligands.

No.	Target	Target Name	Ligand Type	Ligand Count	Decoy Count
1	5HT1A	5-hydroxytryptamine receptor 1A	Agonist	952	37,128
2	5HT1A	5-hydroxytryptamine receptor 1A	Antagonist	506	19,734
3	5HT1D	5-hydroxytryptamine receptor 1D	Agonist	558	21,762
4	5HT1D	5-hydroxytryptamine receptor 1D	Antagonist	315	12,285
5	5HT2A	5-hydroxytryptamine receptor 2A	Antagonist	725	28,275
6	5HT2C	5-hydroxytryptamine receptor 2C	Agonist	209	8151
7	5HT2C	5-hydroxytryptamine receptor 2C	Antagonist	318	12,402
8	5HT4R	5-hydroxytryptamine receptor 4	Agonist	235	9165
9	5HT4R	5-hydroxytryptamine receptor 4	Antagonist	241	9399
10	AA1R	Adenosine receptor A1	Antagonist	280	10,920
11	AA2AR	Adenosine receptor A2a	Antagonist	361	14,079
12	AA2BR	Adenosine receptor A2b	Antagonist	370	14,430
13	ACM1	Muscarinic acetylcholine receptor M1	Agonist	806	31,434
14	ACM3	Muscarinic acetylcholine receptor M3	Antagonist	295	11,505
15	ADA1A	Alpha-1A adrenergic receptor	Antagonist	588	22,932
16	ADA1B	Alpha-1B adrenergic receptor	Antagonist	550	21,450
17	ADA1D	Alpha-1D adrenergic receptor	Antagonist	568	22,152
18	ADA2A	Alpha-2A adrenergic receptor	Antagonist	440	17,160
19	ADA2B	Alpha-2B adrenergic receptor	Antagonist	437	17,043
20	ADA2C	Alpha-2C adrenergic receptor	Antagonist	433	16,887
21	ADRB1	Beta-1 adrenergic receptor	Agonist	209	8151
22	ADRB1	Beta-1 adrenergic receptor	Antagonist	211	8229
23	ADRB2	Beta-2 adrenergic receptor	Agonist	206	8034
24	ADRB2	Beta-2 adrenergic receptor	Antagonist	204	7956
25	ADRB3	Beta-3 adrenergic receptor	Agonist	643	25,077
26	AG2R	Type-1 angiotensin II receptor	Antagonist	1502	58,578
27	CCKAR	Cholecystokinin receptor type A	Antagonist	360	14,040
28	CLTR1	Cysteinyl leukotriene receptor 1	Antagonist	333	12,987
29	DRD2	D2 dopamine receptor	Antagonist	529	20,631
30	DRD3	D3 dopamine receptor	Antagonist	317	12,363
31	DRD4	D4 dopamine receptor	Antagonist	665	25,935
32	EDNRA	Endothelin-1 receptor	Antagonist	676	26,364
33	EDNRB	Endothelin B receptor	Antagonist	561	21,879
34	GASR	Gastrin/cholecystokinin type B receptor	Antagonist	567	22,113
35	HRH3	Histamine H3 receptor	Antagonist	313	12,207
36	LSHR	Lutropin-choriogonadotropic hormone receptor	Antagonist	230	8970
37	NK1R	Substance-P receptor	Antagonist	900	35,100
38	OPRD	Delta-type opioid receptor	Agonist	361	14,079
39	OPRK	Kappa-type opioid receptor	Agonist	284	11,076
40	TA2R	Thromboxane A2 receptor	Antagonist	725	28,275
41	V1AR	Vasopressin V1a receptor	Antagonist	225	8775
42	V1BR	Vasopressin V1b receptor	Antagonist	225	8775

## Supplementary Materials

Supplementary materials can be accessed at: http://www.mdpi.com/1420-3049/21/11/1554/s1.
