# Supplementary material for: Three-Dimensional Biologically Relevant Spectrum (BRS-3D): Shape Similarity Profile Based on PDB Ligands as Molecular Descriptors
Source: Molecules. 2016 Nov 17;21(11):1554. doi: 10.3390/molecules21111554 (PMC6273508; doi:10.3390/molecules21111554)
Supplement: Supplementary file 1 [file molecules-21-01554-s001.pdf]

Supplementary Materials: Three-Dimensional Biologically Relevant Spectrum (BRS-3D): Shape Similarity Profile Based on PDB Ligands as Molecular Descriptor

Ben Hu, Zheng-Kun Kuang, Shi-Yu Feng, Dong Wang, Song-Bing He and De-Xin Kong

Table S1. SVM models of AChE inhibitors based on different sizes of BRCD-3D.

Size of BRCD-3D	CV AUC	Prediction Results of Test Sets			
		Accuracy	Precision	Recall	MCC
BRCD_500	0.972	0.939	0.938	0.895	0.869
BRCD_300	0.970	0.943	0.951	0.895	0.879
BRCD_200	0.967	0.943	0.955	0.891	0.879
BRCD_100	0.959	0.932	0.945	0.870	0.855
BRCD_50	0.947	0.925	0.913	0.882	0.838

The training set consists of 951 AChE inhibitors and 1600 ACD compounds. The test set consists of 238 AChE inhibitors and 399 ACD compounds.

Table S2. SVM models of HIV-1 protease inhibitors based on different size of BRCD-3D.

Size of BRCD-3D	CV AUC	Prediction Results of Test Sets			
		Accuracy	Precision	Recall	MCC
BRCD_500	0.989	0.962	0.969	0.917	0.914
BRCD_300	0.988	0.964	0.984	0.907	0.919
BRCD_200	0.988	0.960	0.955	0.926	0.911
BRCD_100	0.984	0.959	0.959	0.917	0.907
BRCD_50	0.980	0.947	0.943	0.897	0.881

The training set consists of 820 HIV-1 protease inhibitors and 1600 ACD compounds. The test set consists of 204 HIV-1 protease inhibitors and 399 ACD compounds.

Table S3. Enzyme classification of the targets of BRCD-3D ligands.

Enzyme Classification	Count
Oxidoreductases	15
Transferases	103
Hydrolases	122
Lyases	4
Isomerases	7
Ligases	4
Non-enzyme	45
Sum	300

Table S4. SCOP classification of the targets of BRCD-3D ligands.

SCOP Classification	Count
All alpha proteins	23
All beta proteins	63
Alpha and beta proteins (a + b)	63
Alpha and beta proteins (a/b)	46
Small proteins	16
Multi-domain proteins (alpha and beta)	2
Sum	213 [a]

[a] Some targets were without SCOP annotations.

Table S5. The targets types of BRCD-3D ligands.

Class	Sub-Class	Count
Enzyme	Oxidoreductases	15
	Kinase	80
	Other transferases	23
	Protease	90
	Other hydrolases	32
	Lyases	4
	Isomerases	7
	Ligases	4
	**Total Number of Enzyme**	255
Receptor	Nuclear receptor	13
	Ligand-gated ion channel	1
	**Total Number of Receptor**	14
Other class	Transport protein	10
	Cytokine	3
	Contractile protein	2
	Cell adhesion related protein	2
	Cell cycle related protein	1
	Peptide binding protein	1
	Lipid binding protein	1
	Calcium-binding protein	1
	DNA binding protein	1
	RNA binding protein	1
	Growth factor receptor-bound protein	1
	Inhibitor of apoptosis	1
	Translation protein	1
	Lectin	1
	Motor protein	1
	Membrane protein	1
	Chaperone	1
	Unknown function	1
	**Total Number of Other Class**	31

Table S6. Comparison of the three methods handling data imbalance for the 42 GLL/GDD data sets. The CV AUC is 10-fold cross-validation result of the training set. Accuracy, Precision, Recall and MCC are the results of the test set.

Data Sets	CV AUC			Accuracy			Precision			Recall			MCC		
	Weighted	1:39	1:10	Weighted	1:39	1:10	Weighted	1:39	1:10	Weighted	1:39	1:10	Weighted	1:39	1:10
1	0.989	0.988	0.988	0.994	0.994	0.981	0.986	0.993	0.963	0.763	0.768	0.826	0.865	0.870	0.882
2	0.975	0.975	0.974	0.992	0.994	0.974	0.888	0.931	0.883	0.782	0.802	0.822	0.829	0.861	0.838
3	0.988	0.988	0.986	0.993	0.992	0.982	1.000	1.000	0.959	0.703	0.685	0.838	0.835	0.824	0.887
4	0.980	0.979	0.976	0.995	0.996	0.988	0.981	1.000	0.982	0.825	0.825	0.889	0.898	0.906	0.928
5	0.981	0.981	0.978	0.992	0.992	0.974	0.894	0.981	0.956	0.759	0.703	0.745	0.820	0.827	0.830
6	0.983	0.983	0.978	0.986	0.987	0.970	0.721	0.778	0.889	0.738	0.667	0.762	0.722	0.713	0.807
7	0.957	0.956	0.960	0.991	0.991	0.964	1.000	1.000	0.842	0.625	0.625	0.750	0.787	0.787	0.776
8	0.992	0.992	0.989	0.991	0.991	0.980	1.000	1.000	1.000	0.638	0.638	0.787	0.795	0.795	0.878
9	0.993	0.993	0.991	0.997	0.997	0.994	1.000	1.000	0.979	0.875	0.875	0.958	0.933	0.933	0.965
10	0.986	0.986	0.984	0.992	0.992	0.979	0.894	0.915	0.939	0.750	0.768	0.821	0.814	0.834	0.867
11	0.985	0.985	0.984	0.995	0.995	0.981	0.983	0.983	0.953	0.808	0.808	0.836	0.889	0.889	0.883
12	0.984	0.983	0.986	0.993	0.991	0.975	0.894	0.980	0.909	0.797	0.662	0.811	0.841	0.801	0.845
13	0.985	0.984	0.984	0.992	0.993	0.980	0.851	0.927	0.938	0.820	0.789	0.839	0.831	0.851	0.876
14	0.983	0.982	0.978	0.991	0.990	0.968	0.930	0.949	1.000	0.678	0.627	0.644	0.790	0.767	0.789
15	0.983	0.984	0.981	0.993	0.994	0.981	0.968	0.968	0.970	0.763	0.771	0.814	0.856	0.861	0.878
16	0.988	0.987	0.986	0.994	0.994	0.984	0.889	0.977	0.950	0.873	0.782	0.873	0.878	0.871	0.902
17	0.987	0.987	0.982	0.994	0.994	0.982	0.948	0.948	0.950	0.807	0.807	0.842	0.872	0.872	0.885
18	0.953	0.954	0.957	0.991	0.990	0.971	0.983	0.934	0.917	0.648	0.648	0.750	0.794	0.773	0.814
19	0.959	0.958	0.952	0.979	0.991	0.977	0.562	0.924	0.922	0.839	0.701	0.816	0.677	0.801	0.855
20	0.961	0.960	0.957	0.992	0.990	0.968	0.967	0.981	0.938	0.686	0.605	0.698	0.811	0.766	0.793
21	0.995	0.995	0.995	0.992	0.992	0.983	0.912	0.939	0.972	0.738	0.738	0.833	0.816	0.829	0.891
22	0.986	0.986	0.987	0.991	0.991	0.991	0.964	0.964	0.975	0.643	0.643	0.929	0.783	0.783	0.947
23	0.992	0.992	0.989	0.996	0.990	0.989	0.904	0.857	0.974	0.927	0.732	0.902	0.914	0.787	0.931
24	0.990	0.990	0.986	0.995	0.995	0.989	0.971	0.971	0.950	0.829	0.829	0.927	0.895	0.895	0.932
25	0.994	0.994	0.994	0.996	0.996	0.988	0.982	0.982	0.944	0.860	0.853	0.922	0.917	0.913	0.927
26	0.996	0.996	0.996	0.998	0.998	0.994	0.996	0.996	0.990	0.907	0.907	0.940	0.949	0.949	0.961
27	0.986	0.986	0.984	0.993	0.993	0.980	1.000	1.000	0.983	0.722	0.722	0.792	0.847	0.847	0.872
28	0.981	0.981	0.982	0.992	0.992	0.974	0.979	0.979	0.962	0.701	0.701	0.746	0.825	0.825	0.834
29	0.977	0.976	0.976	0.992	0.992	0.975	0.951	0.974	0.914	0.726	0.717	0.812	0.827	0.832	0.843
30	0.982	0.981	0.983	0.993	0.993	0.984	0.941	0.979	0.949	0.750	0.719	0.875	0.837	0.835	0.902
31	0.993	0.993	0.991	0.995	0.995	0.986	0.982	0.982	0.983	0.827	0.827	0.857	0.899	0.899	0.910
32	0.987	0.986	0.984	0.994	0.994	0.984	0.932	0.972	0.959	0.809	0.772	0.860	0.865	0.863	0.900
33	0.986	0.985	0.983	0.993	0.992	0.981	0.902	1.000	0.950	0.814	0.690	0.841	0.853	0.828	0.884
34	0.990	0.990	0.988	0.995	0.995	0.982	0.979	0.979	0.917	0.816	0.816	0.877	0.891	0.891	0.887
35	0.997	0.997	0.997	0.992	0.992	0.981	0.958	0.958	0.963	0.730	0.730	0.825	0.833	0.833	0.882
36	0.990	0.990	0.990	0.989	0.989	0.972	1.000	1.000	0.971	0.543	0.543	0.717	0.733	0.733	0.821
37	0.980	0.980	0.979	0.991	0.991	0.973	0.914	0.975	0.970	0.711	0.639	0.728	0.802	0.785	0.828
38	0.990	0.990	0.987	0.993	0.993	0.989	1.000	1.000	1.000	0.722	0.722	0.875	0.847	0.847	0.930
39	0.990	0.990	0.986	0.990	0.990	0.979	1.000	1.000	0.978	0.596	0.596	0.789	0.768	0.768	0.869
40	0.991	0.991	0.990	0.994	0.994	0.982	0.974	0.982	0.976	0.772	0.759	0.827	0.864	0.860	0.890
41	0.986	0.986	0.983	0.993	0.993	0.980	0.971	0.971	0.973	0.733	0.733	0.800	0.840	0.840	0.872
42	0.983	0.983	0.984	0.992	0.992	0.982	0.969	0.969	1.000	0.689	0.689	0.800	0.813	0.813	0.886

Table S7. Comparison of the Enrichment Factor (EF) of BRS-3D based models and of docking.

Data Sets	BRS-3D [a]		Docking [b]			
	EF_2	EF_{10}	EF_{max}	EF_2	EF_{10}	PDB ID [c]
AA2AR_Antagonist	39.6	9.3	12.9	7.2	3.3	3EML [d]
			32.8	11.7	3.1	3EML [e]
ADRB1_Agonist	36.2	9.5	12.7	10.8	5.2	2Y01
			34.3	14.6	5.7	2Y02
			20.0	8.9	5.9	2Y03
			4.8	4.1	3.9	2Y04
ADRB1_Antagonist	39.0	9.8	5.0	3.1	2.6	2VT4
			3.8	2.9	3.0	2YCW
			6.5	5.2	3.1	2YCZ
ADRB2_Agonist	38.9	10.0	38.2	21.0	7.9	3P0G
ADRB2_Antagonist	38.6	10.0	7.4	5.9	3.5	2RH1
			13.3	7.1	3.9	3NY8
			17.1	5.4	3.6	3NY9
			26.7	6.9	3.5	3NYA
DRD3_Antagonist	37.3	9.7	1.7	0.3	1.5	3PBL

[a] The EFs were computed based on the test set of each data sets; [b] The EFs were computed based on each whole data sets. Data of docking results were originally reported in reference: [1]; [c] The PDB ID of targets which were used in docking process; [d] Excluding crystallographic waters; [e] Including eight crystallographic waters.

Table S8. The prediction performance of model used for HDAC1 inhibitor screening.

Train/Test [a]	CV AUC	Testing Results			
		Accuracy	Precision	Recall	MCC
6536/4301	0.989	0.975	0.716	0.683	0.686

[a] 1737 non-repetitive inhibitors of human HDAC1 (ChEMBL Target ID: ChEMBL325) with definite IC_{50} values were extracted from the ChEMBL compound database (version 17) and used as the positive samples in the SVM model. And 10,000 compounds with similar physicochemical properties to the active compounds were extracted from MDL Drug Data Report (MDDR) and used as the negative sample. From above samples, 1516 HDAC1 inhibitors and 5020 MDDR compounds were selected randomly and used as the training set to build the SVM discriminant model. The remaining compounds were used as the test set to verify the predictive power of model.

Table S9. The subtype selectivity regression models for cannabinoid receptor ligands.

Train/Test	Feature	Q^2	RMSE_CV	R^2	RMSE
1204/301	3 (1%)	0.199	1.313	0.213	1.440
	15 (5%)	0.542	0.991	0.653	0.957
	30 (10%)	0.614	0.916	0.705	0.881
	60 (20%)	0.650	0.867	0.753	0.807
	120 (40%)	0.675	0.838	0.750	0.811
	180 (60%)	0.684	0.823	0.766	0.785
	240 (80%)	0.689	0.820	0.770	0.779
	300 (100%)	0.686	0.829	0.768	0.782

Table S10. The subtype selectivity discriminant models for cannabinoid receptor ligands.

Train/Test	Feature	CV AUC	Sensitivity	Specificity	Precision	Accuracy	MCC
542/135	3 (1%)	0.930	0.763	0.949	0.853	0.896	0.737
	15 (5%)	0.982	0.895	0.990	0.971	0.963	0.908
	30 (10%)	0.985	0.921	1.000	1.000	0.978	0.945
	60 (20%)	0.989	0.868	1.000	1.000	0.963	0.909
	120 (40%)	0.990	0.921	0.990	0.972	0.970	0.926
	180 (60%)	0.992	0.895	1.000	1.000	0.970	0.927
	240 (80%)	0.991	0.921	0.979	0.946	0.963	0.908
	300 (100%)	0.991	0.947	0.990	0.973	0.978	0.945

Table S11. SVM models based on different profiles.

Methods	CV AUC	Prediction Results			
		Accuracy	Precision	Recall	MCC
Dock [a]	0.941	0.882 (563/638)	0.879 (189/215)	0.794 (189/238)	0.746
Dock + Sim_align [b]	0.971	0.922 (587/637)	0.920 (206/224)	0.866 (206/238)	0.831
Dock + Sim_score [c]	0.943	0.901 (573/636)	0.872 (205/235)	0.861 (205/238)	0.788
Sim_align [d]	0.971	0.928 (591/637)	0.903 (215/238)	0.903 (215/238)	0.846

[a] The objective compounds were docked into the binding sites using Surflex-Dock. The docking score array was used as independent variables; [b] The objective compounds were docked into the binding sites using Surflex-Dock. Then, the docked conformation for each target was superimposed rigidly to BRCD-3D ligands using Surflex-Sim. The superimposing score array was used as independent variables; [c] The objective compounds were docked into the binding sites using Surflex-Dock. Then, the similarity scores between the docked conformation and BRCD-3D ligands were calculated with Surflex-Sim without optimization. The similarity score array was used as independent variables; [d] The BRS-3D was calculated using Surflex-Sim as described in MATERIALS AND METHODS section.

Table S12. List of 2D topological descriptors calculated by Dragon.

Descriptor	Meaning	Descriptor	Meaning
ZM1	first Zagreb index M1	S3K	3-path Kier alpha-modified shape index
ZM1V	first Zagreb index by valence vertex degrees	PHI	Kier flexibility index
ZM2	second Zagreb index M2	BLI	Kier benzene-likeliness index
ZM2V	second Zagreb index by valence vertex degrees	PW2	path/walk 2 - Randic shape index
Qindex	Quadratic index	PW3	path/walk 3 - Randic shape index
SNar	Narumi simple topological index (log)	PW4	path/walk 4 - Randic shape index
HNar	Narumi harmonic topological index	PW5	path/walk 5 - Randic shape index
GNar	Narumi geometric topological index	PJI2	2D Petitjean shape index
Xt	Total structure connectivity index	CSI	eccentric connectivity index
Dz	Pogliani index	ECC	eccentricity
Ram	ramification index	AECC	average eccentricity
Pol	polarity number	DECC	eccentric
LPRS	log of product of row sums (PRS)	MDDD	mean distance degree deviation
VDA	average vertex distance degree	UNIP	unipolarity
MSD	mean square distance index (Balaban)	CENT	centralization
SMTI	Schultz Molecular Topological Index (MTI)	VAR	variation
SMTIV	Schultz MTI by valence vertex degrees	BAC	Balaban centric index
GMTI	Gutman Molecular Topological Index	Lop	Lopping centric index
GMTIV	Gutman MTI by valence vertex degrees	ICR	radial centric information index
Xu	Xu index	D/Dr03	distance/detour ring index of order 3
SPI	superpendentic index	D/Dr04	distance/detour ring index of order 4
W	Wiener W index	D/Dr05	distance/detour ring index of order 5
WA	mean Wiener index	D/Dr06	distance/detour ring index of order 6
Har	Harary H index	D/Dr07	distance/detour ring index of order 7
Har2	square reciprocal distance sum index	D/Dr08	distance/detour ring index of order 8
QW	quasi-Wiener index (Kirchhoff number)	D/Dr09	distance/detour ring index of order 9
TI1	first Mohar index TI1	D/Dr10	distance/detour ring index of order 10
TI2	second Mohar index TI2	D/Dr11	distance/detour ring index of order 11
STN	spanning tree number (log)	D/Dr12	distance/detour ring index of order 12
HyDp	hyper-distance-path index	T(N..N)	sum of topological distances between N..N
RHyDp	reciprocal hyper-distance-path index	T(N..O)	sum of topological distances between N..O
w	detour index	T(N..S)	sum of topological distances between N..S
ww	hyper-detour index	T(N..P)	sum of topological distances between N..P
Rww	reciprocal hyper-detour index	T(N..F)	sum of topological distances between N..F
D/D	distance/detour index	T(N..Cl)	sum of topological distances between N..Cl

Wap	all-path Wiener index	T(N..Br)	sum of topological distances between N..Br
WhetZ	Wiener-type index from Z weighted distance matrix (Barysz matrix)	T(O..O)	sum of topological distances between O..O
Whetm	Wiener-type index from mass weighted distance matrix	T(O..S)	sum of topological distances between O..S
Whetv	Wiener-type index from van der Waals weighted distance matrix	T(O..P)	sum of topological distances between O..P
Whete	Wiener-type index from electronegativity weighted distance matrix	T(O..F)	sum of topological distances between O..F
Whetp	Wiener-type index from polarizability weighted distance matrix	T(O..Cl)	sum of topological distances between O..Cl
J	Balaban distance connectivity index	T(O..Br)	sum of topological distances between O..Br
JhetZ	Balaban-type index from Z weighted distance matrix (Barysz matrix)	T(S..S)	sum of topological distances between S..S
Jhetm	Balaban-type index from mass weighted distance matrix	T(S..F)	sum of topological distances between S..F
Jhetv	Balaban-type index from van der Waals weighted distance matrix	T(S..Cl)	sum of topological distances between S..Cl
Jhete	Balaban-type index from electronegativity weighted distance matrix	T(S..Br)	sum of topological distances between S..Br
Jhetp	Balaban-type index from polarizability weighted distance matrix	T(P..Cl)	sum of topological distances between P..Cl
MAXDN	maximal electrotopological negative variation	T(F..F)	sum of topological distances between F..F
MAXDP	maximal electrotopological positive variation	T(F..Cl)	sum of topological distances between F..Cl
DELS	molecular electrotopological variation	T(F..Br)	sum of topological distances between F..Br
TIE	E-state topological parameter	T(Cl..Cl)	sum of topological distances between Cl..Cl
S0K	Kier symmetry index	T(Cl..Br)	sum of topological distances between Cl..Br
S1K	1-path Kier alpha-modified shape index	T(Br..Br)	sum of topological distances between Br..Br
S2K	2-path Kier alpha-modified shape index		

Table S13. List of 3D descriptors calculated by MOE.

Descriptor	Meaning
ASA	Water accessible surface area calculated using a radius of 1.4 Å for the water molecule. A polyhedral representation is used for each atom in calculating the surface area.
dens	Mass density: molecular weight divided by van der Waals volume as calculated in the vol descriptor.
glob	Globularity, or inverse condition number (smallest eigenvalue divided by the largest eigenvalue) of the covariance matrix of atomic coordinates. A value of 1 indicates a perfect sphere while a value of 0 indicates a two- or one-dimensional object.
pmi	Principal moment of inertia.
pmiX	x component of the principal moment of inertia (external coordinates).
pmiY	y component of the principal moment of inertia (external coordinates).
pmiZ	z component of the principal moment of inertia (external coordinates).
pmi1	First diagonal element of diagonalized moment of inertia tensor.
pmi2	Second diagonal element of diagonalized moment of inertia tensor.
pmi3	Third diagonal element of diagonalized moment of inertia tensor.
npr1	Normalized PMI ratio pmi1/pmi3.
npr2	Normalized PMI ratio pmi2/pmi3.
rgyr	Radius of gyration.
std_dim1	Standard dimension 1: the square root of the largest eigenvalue of the covariance matrix of the atomic coordinates. A standard dimension is equivalent to the standard deviation along a principal component axis.
std_dim2	Standard dimension 2: the square root of the second largest eigenvalue of the covariance matrix of the atomic coordinates. A standard dimension is equivalent to the standard deviation along a principal component axis.
std_dim3	Standard dimension 3: the square root of the third largest eigenvalue of the covariance matrix of the atomic coordinates. A standard dimension is equivalent to the standard deviation along a principal component axis.
vol	van der Waals volume calculated using a grid approximation (spacing 0.75 Å).
VSA	van der Waals surface area. A polyhedral representation is used for each atom in calculating the surface area.
vsurf_V	Interaction field volume
vsurf_S	Interaction field surface area
vsurf_R	Surface rugosity
vsurf_G	Surface globularity
vsurf_W*	Hydrophilic volume (8 descriptors, * can take any value from 1 to 8)
vsurf_IW*	Hydrophilic integy moment (8 descriptors, * can take any value from 1 to 8)
vsurf_CW*	Capacity factor (8 descriptors, * can take any value from 1 to 8)
vsurf_Ewmin*	Lowest hydrophilic energy (3 descriptors, * can take any value from 1 to 3)
vsurf_DW*	Contact distances of vsurf_EWmin (3 descriptors, * can take any value among 12, 13 and 23)
vsurf_D*	Hydrophobic volume (8 descriptors, * can take any value from 1 to 8)
vsurf_ID*	Hydrophobic integy moment (8 descriptors, * can take any value from 1 to 8)
vsurf_EDmin*	Lowest hydrophobic energy (3 descriptors, * can take any value from 1 to 8)
vsurf_DD*	Contact distances of vsurf_DDmin (3 descriptors, * can take any value among 12, 13 and 23)
vsurf_HL*	Hydrophilic-Lipophilic (2 descriptors, * can take any value from 1 to 2)
vsurf_A	Amphiphilic moment
vsurf_CP	Critical packing parameter
vsurf_Wp*	Polar volume (8 descriptors, * can take any value from 1 to 8)
vsurf_HB*	H-bond donor capacity (8 descriptors, * can take any value from 1 to 8)

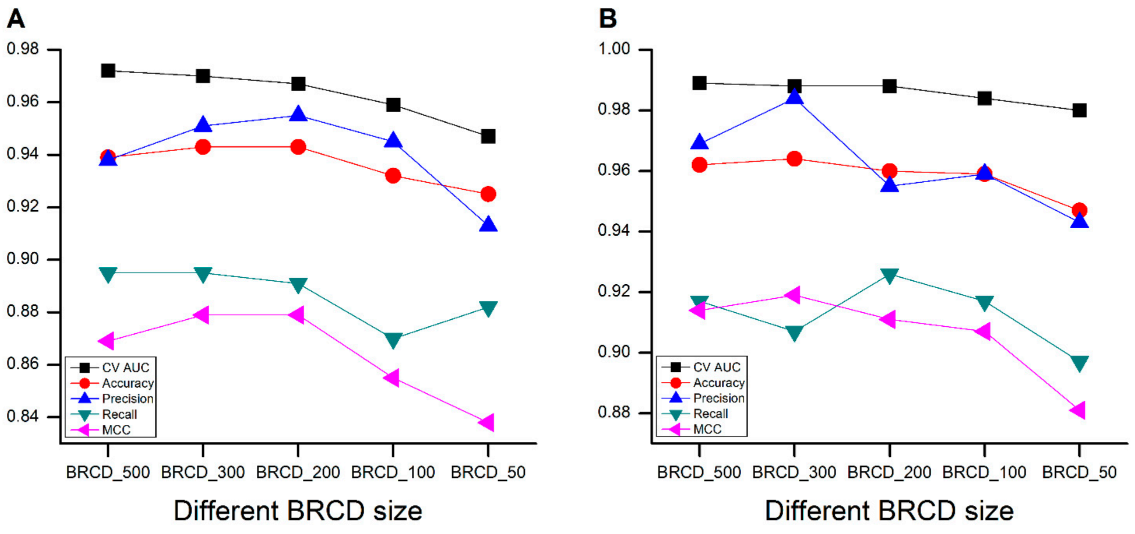

Figure S1. The results of the models developed with different size of BRCD-3D. (**A**) The SVM classification model of AChE inhibitors; (**B**) The SVM classification model of HIV-1 protease inhibitors.

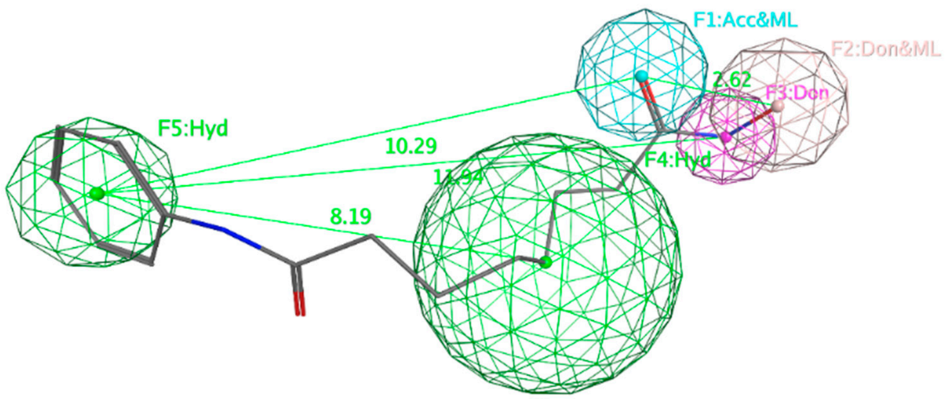

Figure S2. The pharmacophore model based on the conformation of SAHA in 1T69. The pharmacophore model was defined with the pharmacophore query editor in MOE 2009. The feature types and radii are as follows. Feature 1 (F1) is hydrogen bond acceptor and metal ligator (Acc&ML) with radius of 1.3 Å. Feature 2 (F2) is hydrogen bond donor and metal ligator (Don&ML) with radius of 1.3 Å. Feature 3 (F3) is hydrogen bond donor (Don) with radius of 1 Å. Feature 4 (F4) and feature 5 (F5) are hydrophobic centroid (Hyd) with radii of 2.5 and 1.4 Å, respectively. The green numbers represent the distance between two feature centers, and the unit is Å.

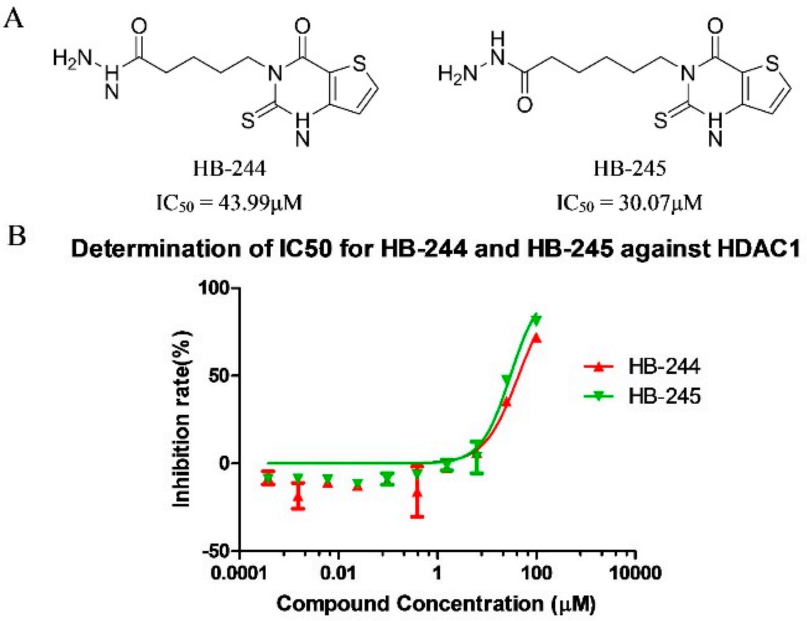

Figure S3. The structures (**A**) of the two active compounds identified with BRS-3D integrated screening protocol and their inhibition curve (**B**) against HDAC1. HDAC1 inhibition assay was tested by the company Medicilon (http://www.medicilon.com/). The enzymatic activity was assessed using a fluorogenic HDAC1 assay kit purchased from BPS Bioscience (San Diego, CA, USA). Briefly, the HDAC1 enzyme was incubated with vehicle or various concentrations of test compounds at 37 °C for 45 min in the presence of an HDAC1 fluorometric substrate. After the HDAC1 assay developer (which produced a fluorophore in the reaction mixture) was added, the fluorescence was measured using a SpectraMax M5 (Molecular Devices) plate reader with excitation at 360 nm and emission at 460 nm. The measured activities were calculated using GraphPad Prism (GraphPad software, San Diego, CA, USA). HDAC assay buffer (Cat: 50031), developer (Cat: 50030), substrate (Cat: 50037), and HDAC 1 (Cat: 50051) were all included in the HDAC1 assay kit. Black opaque 96-well microplates (Cat: 6005270) from Perkin Elmer were used with the SpectraMax M5 plate reader from Molecular Devices.

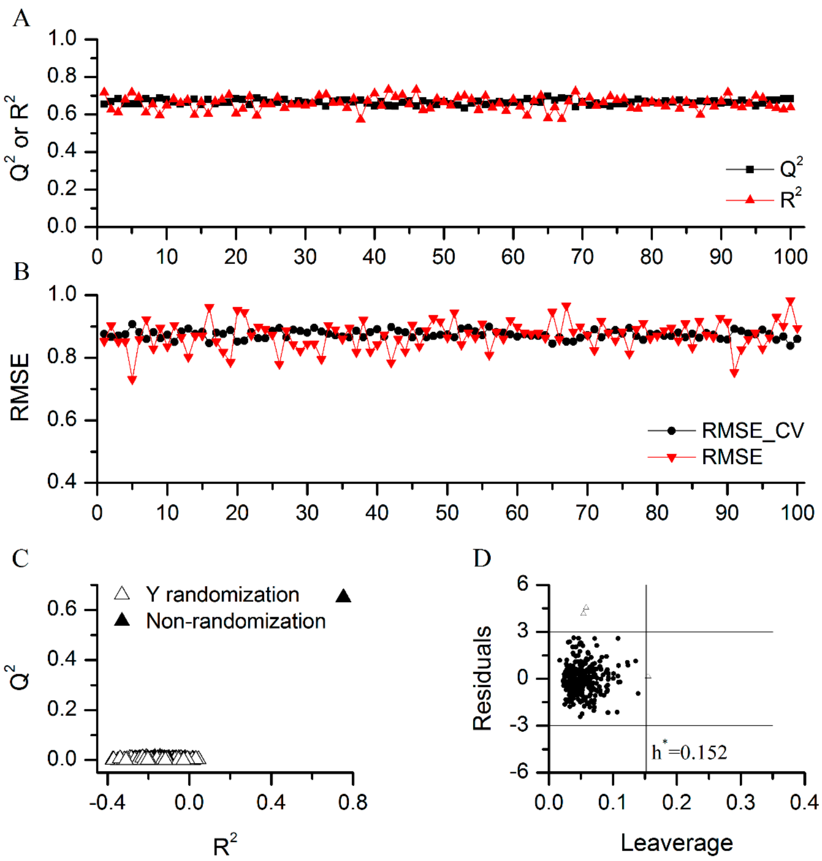

Figure S4. (**A**,**B**) The results of 100 resampling processes; (**C**) The comparison of Y-randomization models and original model; (**D**) The Williams plot for the applicability domain (AD) analysis of models. The resampling process, Y-randomization test and AD analysis were applied for the selectivity regression model with 20% features. In the resampling process, the whole samples were randomly divided into training set and test set for 100 times. For each division, model developed using training set was conducted to predict the test set. The training and test were repeated 100 times, and results were illustrated in (**A**,**B**). The Y-Randomization test was to randomize the response variables (SR values) of training set and develop model to predict the original test set, which was repeated 500 times. Additionally, the AD was quantified by the leverage value (*h*), which were generated by the Hat Matrix (**H**) calculation: $\mathbf{H} = \mathbf{X}(\mathbf{X}^T\mathbf{X})^{-1}\mathbf{X}^T$, where **X** is the descriptor matrix, $\mathbf{X}^T$ is the transpose of **X**, and $(\mathbf{X}^T\mathbf{X})^{-1}$ is the inverse of matrix $(\mathbf{X}^T\mathbf{X})$. The leverages are the diagonal elements of the **H** matrix. A warning leverage (h^*) is generally computed by $h^* = 3p/n$, where p is the number of independent variables plus one and n is the number of compounds in the training set. The triangle points in (**D**) are outside the AD and the prediction for these points may not reliable.

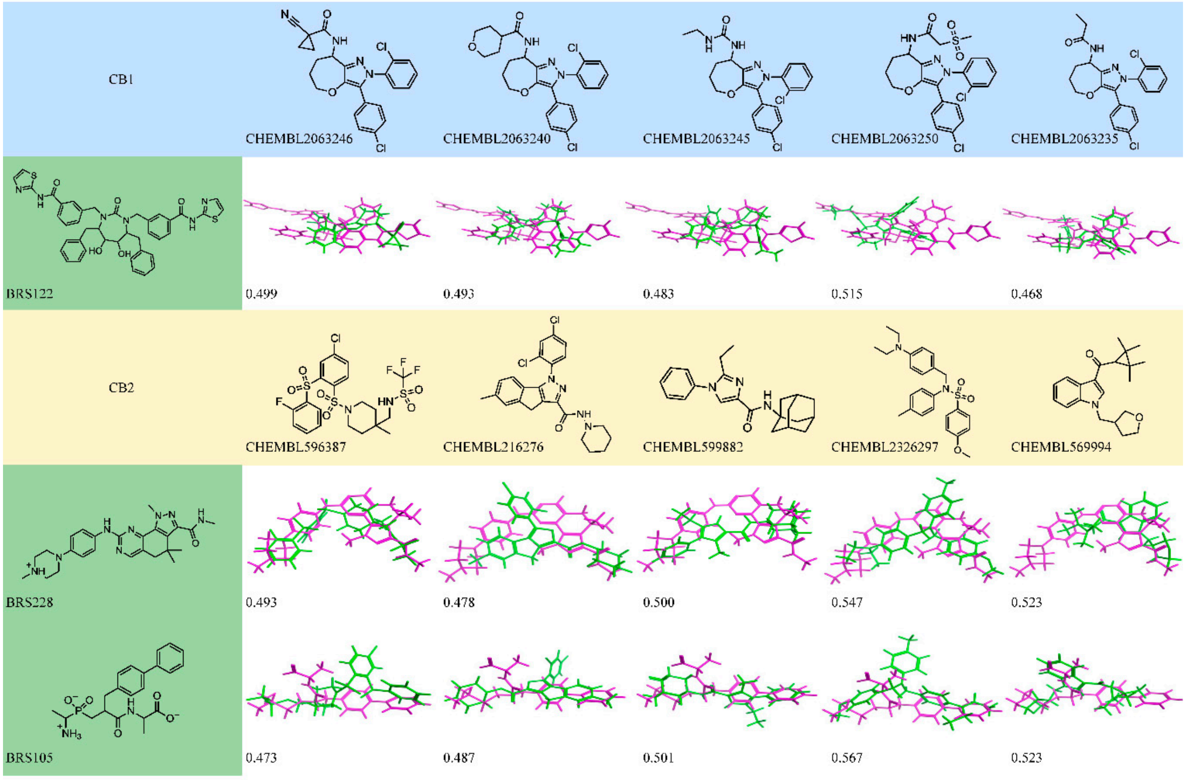

Figure S5. The superimpositions of the 5 most selective compounds of CB_1/CB_2 and corresponding ligands of the most important features. The magenta structures are the ligands of the most important features, and the green structures are the selective compounds of CB_1/CB_2. The values at the left bottom of alignment structures are the superimposition scores. The 2D topological structures of the selective compounds and ligands were dissimilar to each other. However, the 3D shapes of them were similar and these relationships can be detected with BRS-3D analysis of large dataset.

Reference

1. Gatica, E.A.; Cavasotto, C.N. Ligand and Decoy Sets for Docking to G Protein-Coupled Receptors. *J. Chem. Inf. Model.* **2012**, 52, 1–6.
